# 

**Published:** 1917-07-21

**Authors:** 


### CONTENTS.

**The Profession, the Public, and the Politicians** 305
**Are Baby Shows Vulgar and Valueless?** ... 306
**Hospital and Institutional News** ... ... ... 307
The Royal College of Surgeons—Colonel Carter and Sir Victor Horsley—France's Day—The Red Cross in Scotland—Medical Services in Mesopotamia—Medical Education for India—Remedial Baths for Orthopædic Cases—Health of the Salonika Army—The Maternity Unit at Cardiff—What is Distress Due to the War?—The Physique of the U.S. Army—American Red Cross Aid for Russia—The Latest Orthopædic Hospital—The Red Cross in Mesopotamia—Doubling a Saturday Fund Collection—Exempted Tuberculous Patients—Cinema Instruction for Orthopædic Patients—The Wily Shirker—The Dublin Milk Supply—The Health of Munition Workers—Pensioners Who Plead Poverty—Serbians' Gratitude to an Englishwoman—A Foodless Day—A School for Orderlies—Asylum Staffs and Military Service: "A Dangerous Position"—Dental Students at Guy's Hospital—The Military Complaint—This Week's Drug Market.
**On Spiritualism** ... ... ... ... ... ... 313
Dr. Mercier and Sir Oliver Lodge.
**Parliamentary Inquiry on the Medical Examination of Recruits** ... ... ... 314
Some Lay Evidence.
**National Insurance Act Developments...** ... 315
The Future of Medical Benefit.
**Blind, Deaf, Defective, and Epileptic Children** 317
Their Education and Training.
**Has Cotton Grass a Commercial Value?** ... 318
**Asylum Workers' Union** ... ... ... ... 318
**The Matrons' and Sisters' Department** ... 319
The Work of Hospital Almoners.
Round the Hospitals.
The College of Nursing: Second Annual Meeting. Full Report of Speeches—That Uniform Once More!—Ladies' Associations and Auxiliaries—A Glasgow Matron's Career.
**The College of Nursing, Limited** ... ... 323
Nurse Democrats Voice their Problems. Important Questions and Clear Answers.
The Second Annual General Meeting.
**The National Relief Fund** ... ... ... ... 32[illegible]
**Matrons' and Sisters' Appointments** ... ... iv
**The Institutional Worker** ... ... (Suppl.) 1

